# mRNA detection of individual cells with the single cell nanoprobe method compared with *in situ *hybridization

**DOI:** 10.1186/1477-3155-5-7

**Published:** 2007-10-10

**Authors:** Hironori Uehara, Yuji Kunitomi, Atsushi Ikai, Toshiya Osada

**Affiliations:** 1 Department of Life Science, Graduate School of Bioscience and Biotechnology, Tokyo Institute of Technology, Nagatsuta, Midori-ku, Yokohama 226-8501, Japan

## Abstract

**Background:**

The localization of specific mRNA generates cell polarity by controlling the translation sites of specific proteins. Although most of these events depend on differences in gene expression, no method is available to examine time dependent gene expression of individual living cells. *In situ *hybridization (ISH) is a powerful and useful method for detecting the localization of mRNAs, but it does not allow a time dependent analysis of mRNA expression in single living cells because the cells have to be fixed for mRNA detection. To overcome these issues, the extraction of biomolecules such as mRNAs, proteins, and lipids from living cells should be performed without severe damage to the cells. In previous studies, we have reported a single cell nanoprobe (SCN) method to examine gene expression of individual living cells using atomic force microscopy (AFM) without killing the cells.

**Results:**

In order to evaluate the SCN method, we compared the SCN method with *in situ *hybridization (ISH). First, we examined spatial β-actin mRNA expression in single living cells with the SCN method, and then the same cells were subjected to ISH for β-actin mRNA. In the SCN method, quantity of β-actin mRNA were analysed by quantitative PCR, and in ISH we used intensity of ISH as a parameter of concentration of β-actin mRNA. We showed that intensity of ISH is higher; quantity of β-actin mRNA detected by the SCN method increased more.

**Conclusion:**

In this study, we compare the SCN method with the ISH. We examined β-actin mRNA expression in single cells using both methods. We picked up β-actin mRNA from several loci of a single living cell using an AFM nanoprobe, and identical cells were subjected to ISH. The results showed a good correlation between the SCN method and ISH. The SCN method is suitable and reliable to examine mRNAs at medium or higher expression level.

## Background

*In situ *hybridization (ISH) is a powerful molecular tool used to visualize nucleic acids, and it has attributed significantly to the advancement of the study of gene expression in cells and tissues. ISH was invented by two groups in 1969 [1,2]. Around that time, only radioisotope (RI) was available to label nucleic acids. But nowadays, non-RI ISH can be preformed based on synthesis of nucleotides containing certain functional groups and synthesis of a modified oligonucleotide by Digoxigenin (DIG) system [3-6]. Its primary advantage over the Northern blot and reverse transcription polymerase chain reaction (RT-PCR) is its ability to detect localization of specific mRNA to a particular cell or a particular region in a cell. So ISH are applied for bacteria, culture cells, tissue section and whole mount embryo [7-11]. However, ISH cannot examine time-lapse change of identical cells because the cells have to be fixed.

We reported a single cell nanoprobe (SCN) method to examine mRNA expression without killing cells in a previous report [12-14]. In the method, an atomic force microscope (AFM) is used as a manipulator to obtain cell components containing mRNA from the target living cells. AFM has been applied for various biological samples because it can be operated in solution [15-19]. An AFM probe is inserted into the living cells to extract mRNAs. Obtained mRNAs are subjected to RT-PCR and then to nested PCR or quantitative PCR. Since the AFM has high positional and loading force control, extraction of cell components without severe damage to the cells is possible. By using the SCN method, we examined time-lapse mRNA expression change and mRNA localization in single living cells [12-14]. In those studies, we showed that the SCN method has the possibility of compensating for the disadvantages of ISH in the case of the single-cell study.

## Results and discussion

**Figure 1:**
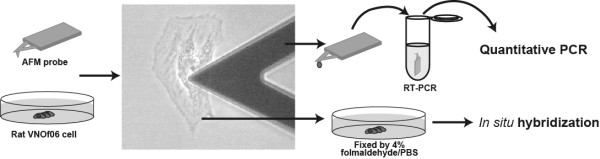
**Experimental overview of the SCN method and ISH**. The AFM probe was inserted into a cell to take mRNAs, and then analyzed with RT-PCR, followed by quantitative PCR. The same cell was fixed by 4%folmaldehyde/PBS and subjected to ISH.

**Figure 2:**
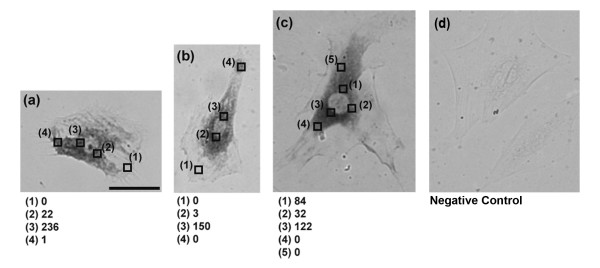
**The results of the SCN method and ISH**. (a-c) Each square indicates the region of the AFM probe insertion. Numbers in lower panels indicate β-actin mRNA quantities detected by the SCN method, and dark intracellular staining indicates distribution of β-actin mRNA detected by ISH. (d) Negative control using sense RNA probe. Scale bar is 50 μm.

The purpose of this study is to evaluate the SCN method and compare it with ISH. Thus it was necessary for us to analyze the same cells with both methods (Fig. 1). First, we picked up mRNAs from single living cells from 3–5 different regions around or far from the nucleus using the SCN method. After the cell components containing mRNA were picked up, the AFM probe that adsorbed the mRNAs was subjected to PCR. The target cell was immediately fixed with 4% formaldehyde/PBS and subjected to ISH. The interval time from first extraction of mRNA to the cell fixation was about 15 minutes. After 30 cycles of RT-PCR, quantitative real-time PCR was performed using 1 μl of RT-PCR reaction buffer as a template. The amounts of initial β-actin mRNA were determined by a standard curve built using β-actin cDNA. Figure 2 shows some examples of ISH and the amounts of β-actin mRNA extracted with the SCN method. Each square (7 × 7 μm) in Fig. 2 indicates the region of insertion with the AFM probe. The number of each square is the order of insertion with the AFM probes. β-actin mRNAs were detected mainly in the vicinity of the nucleus. Usually β-actin mRNA is known to exist mainly in the vicinity of the nucleus, and these results agreed with the results of our previous work and other studies by ISH [13,20]. Alkaline phosphatase activity (dark intracellular staining) corresponded to the distribution of endogenous β-actin mRNA at the time of fixation of the cell. Our ISH results also showed that dark staining was observed around the nucleus predominately, indicating that β-actin mRNAs existed mainly in the vicinity of the nucleus.

In order to analyze the correlation between both methods more precisely, we attempted to standardize and evaluate the ISH results. The darkness of ISH corresponded to β-actin mRNA concentration. But this concentration was not considered to have a linear correlation with the mRNA concentration because it should be considered as absorption of light from a halogen lamp. To analyze the correlation between darkness and β-actin mRNA concentration, we applied a Lambert-Beer-like rule to these results.

[IISH: Intensity of *ISH*] = -Log_10 _(I_n_/I_0_)

**Figure 3:**
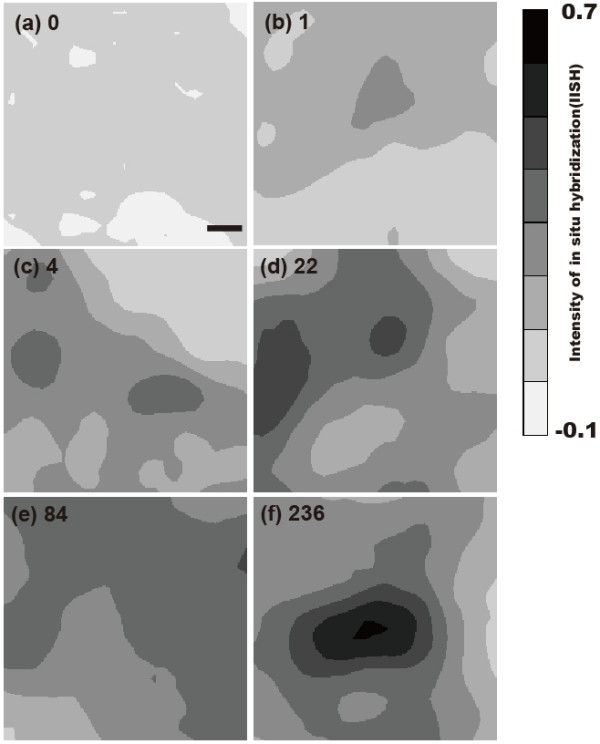
**ISH result in higher resolution**. Black color indicates high intensity of ISH. As black becomes white, the intensity of ISH decreases. The numbers of each figure are the β-actin mRNA quantity detected by the SCN method. Scale bar is 1 μm.

I_0 _is the average of the background intensity. I_n _is the darkness intensity of each point of the cell. In this equation, we considered (I_n_/I_0_) as the transmission. Since the background intensity was stable, we calibrated the darkness of each point on the cell according to the average of the background darkness. Although IISH does not have its own unit, we could compare linearly the ratio between each point. Figure 3 shows high magnification images of IISH results with the amounts of β-actin mRNA picked up by the SCN method. In this figure, IISH was divided into 8 classes within the range of -0.1 and 0.7. The center of each image is the position of the center of the AFM probe inserted. β-actin mRNA was not detected in the region of Fig. 3(a) whose IISH was distributed from -0.1 to 0.1. We could detect a very low β-actin mRNA quantity by the SCN method as shown in Figures 3(b) and 3(c) which show IISHs distributed from 0.1 to 0.4. When IISH was shown to be mainly from 0.2 to 0.5, such as seen in Figures 3(d) and 3(e), more β-actin mRNA was detected by the SCN method. In addition, when IISH was very strong in the center such as shown in Figure 3(f), a number of β-actin mRNAs were detected by the SCN method. In this way, when IISH became higher and the high intensity region became larger, the amounts of β-actin mRNA detected with the SCN method became higher. These results indicated a good relationship between the results of the ISH and the SCN method.

The table summarizes the comparison between the average of IISH and the SCN method. In this table, the averages of IISH were generated from the range of 1.4 × 1.4 μm based on the position inserted by an AFM probe which was centered. We used the AFM probes with square pyramid shapes, whose height, horizontal length and 1/2 corn angles were 3, 4 μm, and 35°, respectively. So if we assume that the AFM probe is inserted into the cell by 1 μm, the range is 1.4 × 1.4 μm. In the table, when the average of IISH showed 0 to 0.1, β-actin mRNA was not detected by the SCN method. When the average of IISH was 0.1 to 0.25, β-actin mRNA was detected by the SCN method in low probability (33%) and low quantity. When the average of IISH was over 0.25, the probability of β-actin mRNA detection was 100%. However, the average quantities of β-actin mRNA detected by the SCN method were 50 and 120 molecules within the range of 0.25 to 0.4 and over 0.4, respectively. Based on this, as IISH increased more, the probability and quantity of β-actin mRNA detected by the SCN method increased more. These results indicated a proportional relation between the results of the ISH method and the SCN method.

**Figure 4:**
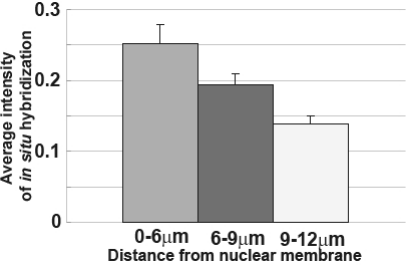
**Relation between ISH intensity and the distance from nuclear membrane**. The average of the ISH intensity was calculated according to the distance from the nuclear membrane. The detection probability of β-actin mRNA with the SCN method changed according to the distance from the nuclear membrane. As the distance from the nuclear membrane became greater, fewer positive results were obtained with the SCN method.

Previously, we showed that detection probability of β-actin mRNA by the SCN method changed according to the distance from a nuclear membrane [13]. When the region was 0–6 μm from the nuclear membrane, the probability of detecting β-actin mRNA was 100%. As the distance from the nuclear membrane became greater, the probability decreased. In the region 6–9 μm from the nuclear membrane, the probability was 61.5%, and in the region 9–18 μm away from the membrane, it was 11.1%. To compare these previous results with the ISH results, we calculated the average of IISH along with the distance from the nuclear membrane. In the region of 0–6 μm from the nuclear membrane, the average of IISH was 0.25, as the distance increased more, the average of IISH decreased (Figure 4). This tendency was similar with the distance dependency of the detection possibility in the SCN method. In the region of IISH > 0.25, we could detect β-actin mRNA with the SCN method at 100% probability (Table 1). This also shows that a good correlation between ISH and the SCN method, and the SCN is suitable to detect mRNA at medium or above expression.

**Table 1:** Comparison between single cell nanoprobe method and ISH result (± S.D)

ISH intensity		0–0.1	0.1–0.25	0.25–0.4	0.4-
Single cell nanoprobe method	β-actin mRNA detection probability	0%(n = 5)	33%(n = 6)	100%(n = 7)	100%(n = 3)
	Average number of detected β-actin mRNA	0	5(± 5)	50(± 20)	120(± 65)

## Conclusion

We showed the correlation between ISH and the SCN method. The SCN method can examine time-dependent mRNA expression of single living cells, but it is limited to the analysis of the fine localization of mRNA in the cells. ISH can examine mRNA expression of the whole cells with higher resolution, but time-lapse analysis cannot be done. Besides, the SCN method is suitable and reliable to examine mRNAs at medium or higher expression level. By using both methods, more accurate information about mRNA expression of single cells is available.

## Methods

### Preparation of cells

Rat fibroblast-like VNOf06 cells derived from the vomeronasal organ [21] were grown in 35 mm Petri dishes in Dulbecco's minimum essential medium (DMEM)/F12 supplemented with 100 U/mL penicillin, 100 μg/mL streptomycin, and 10% heat-inactivated fetal bovine serum (FBS). The cells were washed three times with DMEM/F12 without FBS and used for the AFM experiments.

### The single cell nanoprobe (SCN) method

The details of the SCN method have been described in previous studies [12-14].

Briefly, the AFM probe (NP, Digital Instruments, Santa Barbara, CA) was positioned onto a target region of cells under the observation of an inverted phase-contrast microscope. The AFM probe was then inserted into the target cell using the step motor of the AFM (NVB-100, Olympus, Inc.), and held for about 30 s to allow the AFM probe to bind the cell components containing mRNA with physical adsorption. The AFM probe was lifted off the cell and placed into a PCR tube.

### PCR

The reagents and primers of RT-PCR and quantitative PCR were used as previously described [12,13]. RT-PCR was performed with a one-step RT-PCR kit (Qiagen, Valencia, CA). First-strand cDNA synthesis was performed at 50°C for 30 min, at which time the reaction was heated to 95°C for 15 min to activate HotStrTaq DNA polymerase. The amplification reaction was carried out for 30 cycles, and each cycle was 94°C for 45 s, 55°C for 45 s, and 72°C for 1 min, followed by a final 10 min elongation at 72°C. Quantitative PCR was performed with an Applied Biosystems Prism 7000 and the SYBR Green 1 PCR Mastermix (Qiagen, CA, USA) following previous studies [12,13].

### ISH for β-actin mRNA of single cells

#### Digoxigenin (DIG) labeled RNA probe preparation

β-actin cDNA [224–987 bp] was prepared by RT-PCR. β-actin cDNA was inserted into pGEM^(R)^-T Easy vector (promega), and subcloned. The direction of the inserted cDNA was examined by restriction enzyme and by its sequence. Antisense DIG-labeled RNA probe was prepared by SP6 and T7 RNA polymerase (stratagene) and 10 × DIG labeling mix (Roche). The efficiency of DIG labeling was examined by dot-blotting.

#### Cell preparation for ISH

After picking up mRNA by the SCN method, the cells were washed by PBS 3 times and fixed in 4% paraformaldehyde(PFA)/PBS for 30 min. From this point, all treatments were performed under RNase free condition. After PBS washing, the cells were treated by 1 μg/ml proteinase K (Invitrogen) for 5 min at 37°C, washed in PBS, refixed in 4% PFA/PBS for 10 min at RT, neutralized in 0.2% glycine/PBS for 2 min, 0.2 N HCl at RT for 20 min and washed with PBS two times.

#### Hybridization and detection by alkaline phosphatase reaction

Hybridization solution (60% formamide (deionized), 2 × SSC (1 × SSC is 150 mM NaCl, 15 mM), 10 mM EDTA, 25 mM NaH_2_PO_4_, 5% dextran sulfate and RNA probe (added before use)) was add to the cells described above and incubated overnight at 55°C. The RNA probe concentration was determined before the experiment and was adjusted to be 0.1 ng/μl. After overnight incubation, the cells were washed in the following order: 5 × SSC/50% formamide 30 min 50°C two times, TNE buffer 5 min (10 mM Tris, 0.5 M NaCl, 1 mM EDTA pH7.5), 20 g/ml RNase/TNE buffer 30 min 37°C, 2 × SSC 30 min 50°C two times, 0.2 × SSC 30 min 50°C two times, blocking solution (1% Blocking Reagent (Roche)/TBS (0.1 M Tris-HCl pH7.5, 0.15 M NaCl)) 30 min. The cells were then incubated in anti-DIG Fab fragment(Roche) diluted 1:500 with blocking solution for 60 min. After washing with TNT buffer (0.2% Tween20/TBS) 15 min two times and AP buffer (0.1 M Tris-HCl pH9.5, 0.1 M NaCl, 50 mM MgCl_2_), the cells were stained by DIG Nucleic Acid Detection kit (Roche) for 6 hours using alkaline phosphatase reaction of NBT/BCIP. After PBS washing, the cells were embedded in PermaFluor Mountant Medium (Thermo, USA) and obsreved by a phase-contrast microscope and a bright-field microscope.

## Competing interests

The author(s) declare that they have no competing interests.

## Authors' contributions

HU conceived of the study and drafted the manuscript, and carried out PCR and AFM. YK carried out ISH. AI and TO participated in the design of the study and coordination. All authors read and approved the final manuscript.
